# Drug Repositioning for Noonan and LEOPARD Syndromes by Integrating Transcriptomics With a Structure-Based Approach

**DOI:** 10.3389/fphar.2020.00927

**Published:** 2020-06-26

**Authors:** Liyuan Zhu, Ruth Roberts, Ruili Huang, Jinghua Zhao, Menghang Xia, Brian Delavan, Mike Mikailov, Weida Tong, Zhichao Liu

**Affiliations:** ^1^ National Center for Toxicological Research, U.S. Food and Drug Administration, Jefferson, AR, United States; ^2^ Department of Drug Safety, ApconiX, Alderley Edge, United Kingdom; ^3^ Department of Biosciences, University of Birmingham, Birmingham, United Kingdom; ^4^ National Center for Advancing Translational Sciences, National Institutes of Health, Rockville, MD, United States; ^5^ Joint Bioinformatics Graduate Program, University of Arkansas at Little Rock and University of Arkansas for Medical Sciences, Little Rock, AR, United States; ^6^ Office of Science and Engineering Labs, Center for Devices and Radiological Health, U.S. Food and Drug Administration, Silver Spring, MD, United States

**Keywords:** transcriptomic profiles, molecular docking, induced pluripotent stem cell, drug repositioning, rare diseases

## Abstract

Noonan and LEOPARD syndromes (NS and LS) belong to a group of related disorders called RASopathies characterized by abnormalities of multiple organs and systems including hypertrophic cardiomyopathy and dysmorphic facial features. There are no approved drugs for these two rare diseases, but it is known that a missense mutation in PTPN11 genes is associated with approximately 50% and 70% of NS and LS cases, respectively. In this study, we implemented a hybrid computational drug repositioning framework by integrating transcriptomic and structure-based approaches to explore potential treatment options for NS and LS. Specifically, disease signatures were derived from the transcriptomic profiles of human induced pluripotent stem cells (iPSCs) from NS and LS patients and reverse correlated to drug transcriptomic signatures from CMap and L1000 projects on the basis that if disease and drug transcriptomic signatures are reversely correlated, the drug has the potential to treat that disease. The compounds that were ranked top based on their transcriptomic profiles were docked to mutated and wild-type 3D structures of PTPN11 by an adjusted Induced Fit Docking (IFD) protocol. In addition, we prioritized repositioned candidates for NS and LS by a consensus ranking strategy. Network analysis and phenotypic anchoring of the transcriptomic data could discriminate the two diseases at the molecular level. Furthermore, the adjusted IFD protocol was able to recapitulate the binding specificity of potential drug candidates to mutated 3D structures, revealing the relevant amino acids. Importantly, a list of potential drug candidates for repositioning was identified including 61 for NS and 43 for LS and was further verified from literature reports and on-going clinical trials. Altogether, this hybrid computational drug repositioning approach has highlighted a number of drug candidates for NS and LS and could be applied to identifying drug candidates for other diseases as well.

## Introduction

Noonan syndrome (NS) and LEOPARD syndrome [LS, also called Noonan syndrome with multiple lentigines (NSML)] are autosomal dominant disorders with overlapping phenotypic characteristics including hypertrophic cardiomyopathy, short stature, pectus deformity, and dysmorphic facial features (Tartaglia and Gelb, 2005). NS has an estimated prevalence of 1 in 1000~2500. The exact prevalence of LS is unknown and approximately 200 cases have been reported globally (https://ghr.nlm.nih.gov/condition/noonan-syndrome-with-multiple-lentigines#statistics) (Sarkozy et al., 2008). The missense mutation in PTPN11 genes, resulting in a gain-of-function of the non-receptor protein tyrosine phosphatase SHP-2 protein causes approximately 50% of NS cases. Causative mutations in other genes (e.g., SOS1, RIT1, and KRAS) involved in RAS-MAPK pathways have also been identified in a small portion of patients with NS (Schubbert et al., 2006; Pandit et al., 2007; Kouz et al., 2016). Mutations in PTPN11 also causes approximately 70% of LS. Considerable efforts have been made to uncover the molecular basis of the two diseases (Tartaglia et al., 2010). The current management for LS and NS mainly relies on treating the phenotypic presentation of a specific organ or system based on the individual patient (Romano et al., 2010). No drugs have been approved for treating NS or LS by the U.S. Food and Drug Administration (FDA).

Drug repositioning is a well-established approach to providing quicker, safer, and more affordable drugs to fulfill the unmet medical need for rare disease therapies (Ekins et al., 2011; Liu et al., 2016; Delavan et al., 2018). Advances in genomics and bioengineering provide unprecedented opportunities to uncover the underlying mechanisms of diseases where there may be accumulated data profiles that represent the different aspects of biological complexity. These data profiles also enable *in silico* drug repositioning as a parallel approach to exploring potential opportunities for rare disease treatment (Butte et al., 2015; Jia et al., 2018). To date, approximately 1,000 crystal structures for rare disease-associated proteins have been made available *via* The Research Collaboration for Structural Bioinformatics Protein Data Bank (RCSB PDB) (Yang et al., 2018), stimulating research in molecular docking-based drug repositioning. For example, Govindaraj et al. (2018) carried out molecular docking-based virtual screening with optimized drug-binding sites generated by the *e*MatchSite to systematically assess opportunities to repurpose approved drugs for 980 rare diseases.

Novel high throughput screening platforms such as LINCS1000 (Subramanian et al., 2017) and TempO-Seq™ (Liu et al., 2019), combined with the falling cost of next-generation sequencing (NGS), have resulted in transcriptomics data of more than 30,000 compounds being publicly available. Some initial efforts have been made to apply these transcriptomics data sets to seek repositioning candidates for rare diseases. For example, Dudley et al. (2011) comprehensively compared disease gene signatures to drug transcriptomic signatures from CMap to suggest the potential reuse of the anticonvulsant topiramate for inflammatory bowel disease (IBD) treatment. Also, some other approaches such as information retrieval or text mining (Chen and Altman, 2017), miRNA transcription factor feed-forward loops (Liu et al., 2014), and high throughput or high content screening assays (Bellomo et al., 2017) have also been applied to drug repositioning of rare diseases.

Due to our incomplete knowledge of rare diseases, the selection of “fit-for-purpose” *in silico* drug repositioning approaches varies among different rare diseases based on data availability (Delavan et al., 2018). Progress in structural chemistry makes mutated protein structures of NS and LS such as PTPN11 available, enabling the implementation of molecular docking approaches to look for potential repositioning candidates. Furthermore, human induced pluripotent stem cells (iPSCs) derived directly from patient cells hold great promise in mimicking the complex pathogenesis of diseases. Some transcriptomic profiles of NS and LS have been generated using human iPSCs, which provides further information in support of *in silico* drug repositioning. Transcriptomics-based drug repositioning can produce a comprehensive picture of gene perturbation by experimental compounds to assist in identifying different disease-associated biological processes. However, this approach cannot point directly to a therapeutic target for further investigation. Molecular docking aims to rank the binding affinity of tested molecules on the potential therapeutic target, offering a complementary strategy for transcriptomics-based drug repositioning. However, to the best of our knowledge, there are as yet no approaches described that integrate transcriptomics and molecular docking to explore treatment opportunities for NS and LS.

In this study, we implemented a hybrid approach that combines transcriptomics with molecular docking to explore potential repositioned candidates the treatment of NS and LS. First, transcriptomic profiles generated in human iPSC from NS and LS patients with PTPN11 mutations were employed to generate diseases signatures and were further compared using functional and network analysis. Second, the repositioned candidates for NS and LS were enriched by reverse correlation with drug signatures derived from LINCS1000 and CMap data sets. Third, the enriched repositioned candidates were further prioritized using molecular docking approaches. Finally, the promising repositioned candidates for NS and LS were verified from on-going clinical trials and literature reports.

## Results

### Transcriptomic Signatures of Noonan and LEOPARD Syndrome

**Figure 1:**
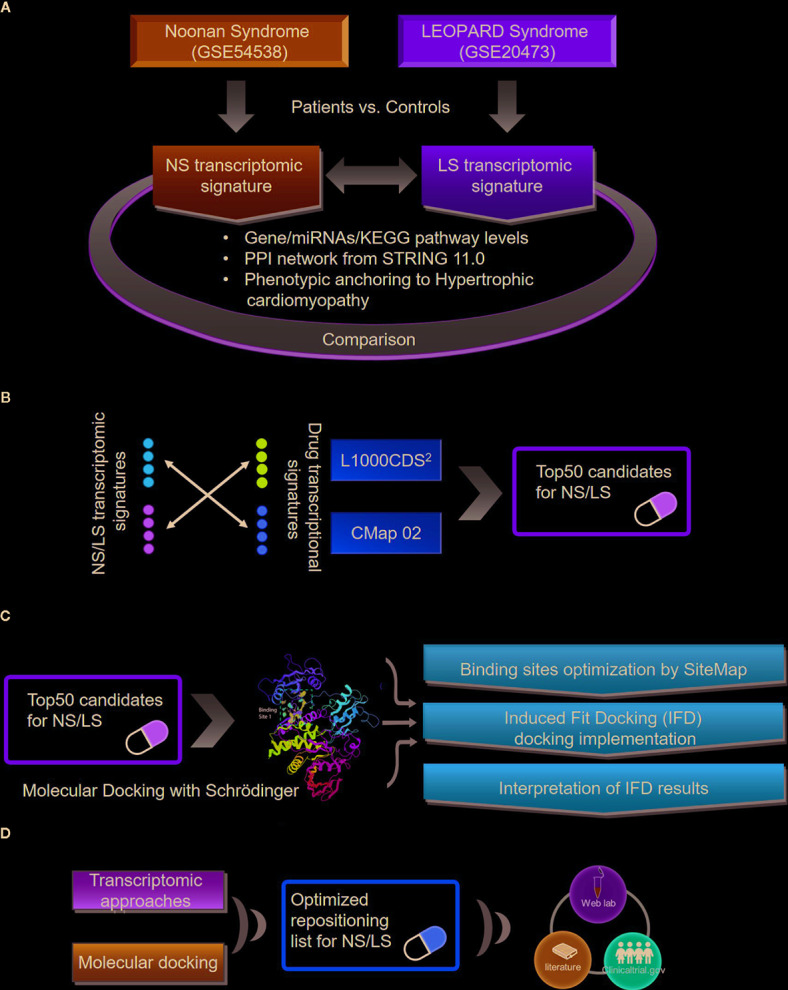
An outline of workflow **(A)** Transcriptomic signatures of Noonan and LEOPARD syndrome. **(B)** Repositioning candidates enriched by transcriptomic approaches. **(C)** Molecular docking for repositioning candidates enriched by transcriptomic approaches. **(D)** Optimization of repositioning candidates by a hybrid strategy.

An outline of the study is illustrated in **Figure 1**, and a summary for transcriptomic data sets of Noonan syndrome (NS), LEOPARD syndrome (LS), and hypertrophic cardiomyopathy (HCM) are given in **Table 1**. Initially, the NS and LS transcriptomic signatures were generated. We used two microarray data sets generated from human iPSC lines for NS (e.g., GEO accession number: GSE54538) and LS (e.g., GEO accession number: GSE20473), respectively. The NS and LS transcriptomic signatures were calculated using patient samples versus their matched controls. The top 250 genes based on ranked fold change values were extracted as NS and LS transcriptomic signatures (see **Supplementary Table S1**).

**Table 1:** Transcriptomic data information for Noonan syndrome, LEOPARD syndrome, and hypertrophic cardiomyopathy (HCM).

GEO accession numbers	Tissue type	Platform	Sample information
**Noonan syndrome (NS)**			
**GSE54538**	Human iPSC line	Illumina HumanHT-12 V4.0 expression beadchip	• 2 patient samples: 1 NS patient with E76D mutation in PTPN11; 2 biological duplicates from human iPSC line • 4 control samples: 1 human embryonic stem cell sample, and 3 induced pluripotent stem cell samples from healthy people
**LEOPARD syndrome (LS)**			
**GSE20473**	Human iPSC line	Affymetrix Human Exon 1.0 ST Array	• 2 patient samples: 2 LS patients with T468M mutation in PTPN11; each patient with 2 biological duplicates from human iPSC line. • 2 control samples: 1 HES2 human embryonic stem cell sample, and 1 induced pluripotent stem cell sample from healthy people
**Hypertrophic cardiomyopathy (HCM)**			
**GSE89714**	Human hypertrophic heart tissue	Illumina HiSeq 2000	• 5 patient samples: patients previously diagnosed with hypertrophic cardiomyopathy, undergoing septal myectomy surgery. • 4 Control samples: normal heart donor left ventricles
**GSE68316**	Human myocardial tissues	CapitalBio Human LncRNA Microarray v2.0	• 7 patient samples: 7 HCM patients • 5 Control samples: 5 disease-free individuals
**GSE36961**	Human surgical myectomy tissue	Illumina HumanHT-12 V3.0 expression beadchip	• 106 patient samples: 106 HCM patients • 39 Control samples: 39 healthy donors
**GSE32453**	Human cardiac myectomy tissue	Illumina humanRef-8 v2.0 expression beadchip	• 8 male patients: hypertrophic obstructive cardiomyopathy (HOCM) • 5 controls: people without cardiac disease

**Figure 2:**
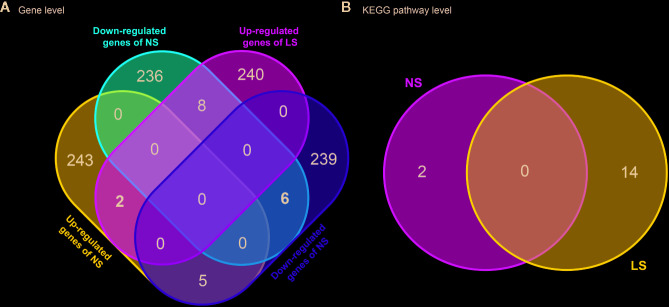
Venn diagram of transcriptomic signatures and related KEGG pathways between Noonan syndrome (NS) and LEPOARD syndrome (LS). **(A)** common genes of up- or down- regulated genes of NS and LS; **(B)** common KEGG pathways enriched by NS and LS transcriptomic signatures.

NS and LS have much in common regarding their phenotypic characters and etiology. To investigate whether the transcriptomic signatures could be used to distinguish between NS and LS, we compared NS and LS signatures at the gene and pathway levels. There was very little overlap (less than 0.1%) between NS and LS transcriptomic signatures, indicating the power of transcriptomic profiles to differentiate between NS from LS (**Figure 2A**). Two common up-regulated genes (*LGALS1* and *PHLDA1*) and six common down-regulated genes (*PRKCB*, *ZIC3*, *PGAM1*, *PMEL*, *FRAT2*, and *MT1G*) were found in the transcriptomic signatures of NS and LS. An analysis of Kyoto Encyclopedia of Genes and Genomes (KEGG) pathways highlighted two over-represented KEGG for NS included Ribosome (KEGG id: hsa03010) and Biosynthesis of antibiotics (KEGG id: hsa01130). Meanwhile, a total of 14 KEGG pathways were over-represented for LS, which consisted of several cardiovascular-related pathways such as Hypertrophic cardiomyopathy (KEGG id: hsa05410) and some cancer-related pathways such as PI3K-Akt signaling pathway (KEGG id: hsa04151) (**Figure 2B** and **Supplementary Table S2**). Similarly, no overlapping pathways were found between NS and LS.

**Figure 3:**
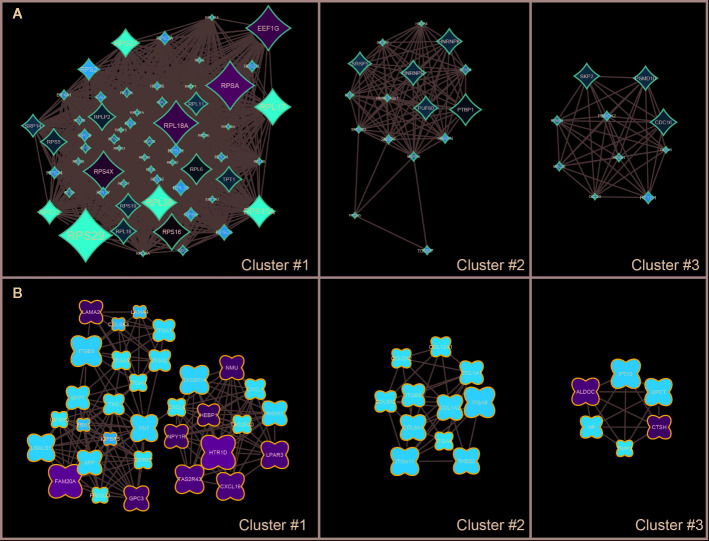
Sub clusters of protein-protein interaction (PPI) networks of Noonan syndrome (NS) and LEPOARD syndrome (LS) transcriptomic signatures by Cytoscape MCODE plugin. **(A)** Top three clusters of NS-related PPI networks based on MCODE enrichment scores; **(B)** Top three clusters of LS-related PPI networks based on MCODE enrichment scores.

The results of the KEGG Pathway analysis were further confirmed *via* a network analysis of NS and LS transcriptomic signatures and protein and protein interactions (PPIs) from the STRING database. The top three clusters of PPIs were extracted using Cytoscape (plugin MCODE), and the over-represented KEGG pathways were enriched using the Database for Annotation, Visualization and Integrated Discovery (DAVID) (**Figure 3** and **Supplementary Table S3**). For NS, the only enriched KEGG pathway was Ribosome (KEGG id: hsa03010). For LS, the enriched pathways were Hypertrophic cardiomyopathy (KEGG id: hsa05410) and PI3K-Akt signaling pathway (KEGG id: hsa04151). These results are consistent with the KEGG pathways enriched with the whole transcriptomic signatures.

The concordance between the NS and LS signatures was also compared at the miRNA level. Specifically, the related miRNAs for NS and LS signatures were obtained by using a consensus of 12 miRNA target prediction algorithms (**Supplementary Table S4**). The concordance between LS and NS was dramatically increased at the miRNA level (**Supplementary Figure S1**); there was a total of 118 and 81 up- and down-regulated miRNAs shared between NS and LS, respectively. This improved concordance indicates that the regulatory mechanisms of the two diseases are interrelated.

The cardiac system is one of the organs most affected by NS and LS. Cardiac disorders such as HCM were present in approximately 20% of NS and LS patients. The pathway analysis indicated that the LS patients had more characteristic features of HCM compared with NS patients. To further understand the relationship between HCM and NS and LS, we extracted HCM-related gene signatures from four transcriptomic profiling data sets (i.e., GSE89714, GSE68316, GSE36961, and GSE32453) and compared them with the NS and LS signatures. There was little overlap of genes among the different studies, possibly due to the high heterogenicity of HCM patients. The number of overlapping genes with HCM studies were 26 (10 up-regulated and 16 down-regulated) and 28 (16 up-regulated and 12 down-regulated) for NS and LS, respectively (**Supplementary Figure S2**).

### Enrichment of Repositioning Candidates by Transcriptomics-Based Approaches

Repositioning candidates were enriched by using two large drug transcriptomic data sets including CMap02 and LINCS 1000 (L1000) (Lamb et al., 2006; Subramanian et al., 2017). The rationale behind transcriptomic-based drug repositioning is if the disease and drug transcriptomic signatures are reversely correlated, the drug has the potential to treat that disease (Iorio et al., 2013). We employed the L1000CDS^2^ web-based tool to enrich repositioning candidates for NS and LS, respectively. The top 50 candidates with highest enrichment scores (e.g., cosine similarity) for each disease were extracted. We noticed that some repositioning candidates were enriched across multiple assay conditions. For example, CGP-60474, an inhibitor of a cyclin-dependent kinase, was identified as a potential treatment for NS based on its transcriptomic profiles in 12 different cell line/concentration/duration combinations. Repositioning candidates were also identified using CMap02 data sets. We collapsed these drug transcriptomic profiles generated under different assay conditions such as cell line/concentration/duration into one prototype ranking list (PRL) to generate its signature. The top 50 repositioning candidates were prioritized based on ranked GSEA scores for NS and LS, respectively. Together, we obtained 74 repositioning candidates for NS and LS from L1000 and CMap data sets, respectively (see **Supplementary Table S5**).

**Figure 4:**
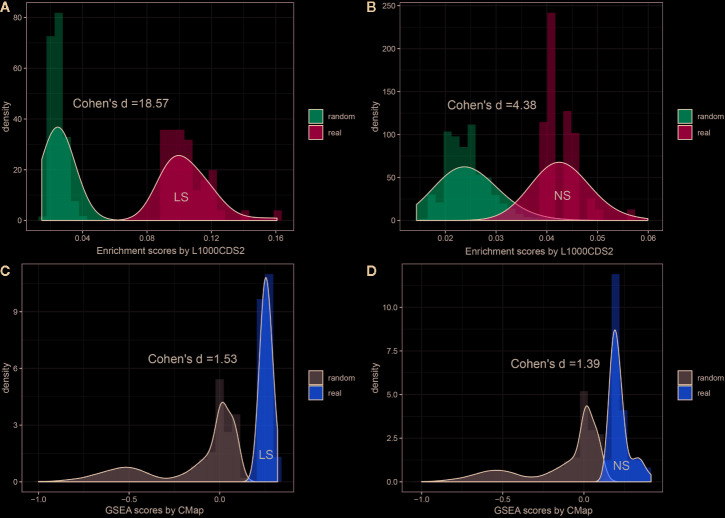
Permutation test of enriched repositioning candidates by using transcriptomic approach. **(A, B)** distribution of enrichment scores generated using NS and LS transcriptomic signatures (shown in blue) and pseudo disease signatures (shown in red) against the L1000 dataset; **(C, D)** distribution of enrichment scores generated using NS and LS transcriptomic signatures (shown in yellow) and pseudo disease signatures (shown in gray) against CMap dataset.

To investigate whether the enriched repositioning candidates were better by chance, we conducted a random test by using a pseudo disease signature to query the two drug transcriptomics datasets. The process was repeated 50,000 times. **Figure 4** illustrates the distribution of enrichment scores for real and pseudo disease signatures. The Cohen's d values with Hedges correction were calculated for assessing the effect sizes between the actual and pseudo distribution. All the Cohen's d values were more than 1.2, considered a very large effect size (Sawilowsky, 2009). Also, the unpaired student t-test was used, and all *p* values were less than 2.2×10^-16^. Both statistical measures indicated that all the enriched repositioning candidates were not attributable to chance.

**Figure 5:**
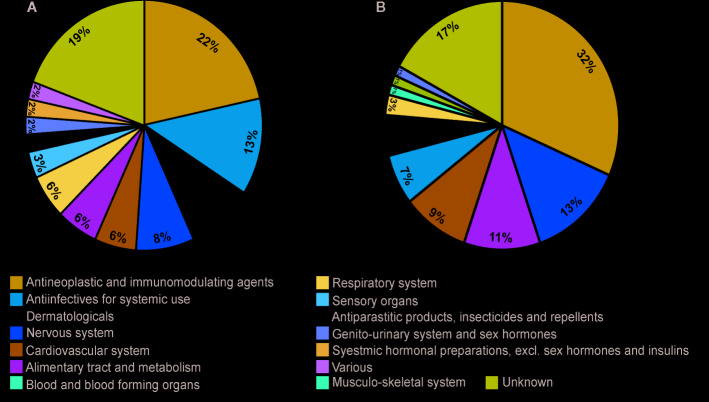
Pie charts of therapeutic categories of enriched repositioning candidates by using transcriptomic approach. **(A)** Therapeutic categories of repositioning candidates for Noonan syndrome; **(B)** Therapeutic categories of repositioning candidates for LEPAROD syndrome.

The therapeutic class distribution of enriched repositioning candidates is shown in **Figure 5**. Repositioning candidates covered more than ten different therapeutic categories as defined by the first level of the WHO Anatomical Therapeutic Chemical (ATC) Classification System. Of them, **L**-Antineoplastic and immunomodulating agents dominated all the therapeutic classes, corresponding to 22% and 32% of repositioning candidates for NS and LS, respectively. It was interesting that 11 repositioning candidates for NS were from **J**-Antiinfectives for systemic use and 10 for LS belonged to **N**-Nervous system, highlighting the divergence of the underlying mechanisms of the two diseases. It was notable that the 8 dermatological drugs were enriched for NS, perhaps correlating with facial deformaties in NS patients.

### Molecular Docking for Enriched Repositioning Candidates

#### Binding Site Identification

**Figure 6:**
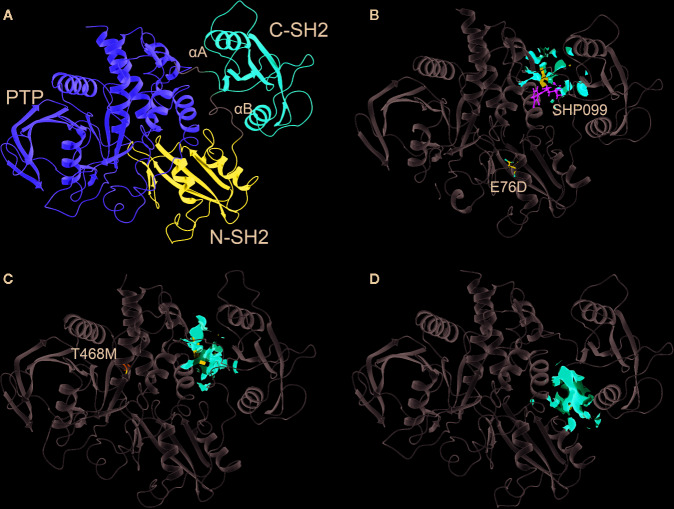
Optimized binding sites of 3D SHP2 protein structures of Noonan syndrome (NS), LEPOARD syndrome (LS) and wild type by using Schrodinger SiteMap module. **(A)** Domain information of SHP2; **(B)** Predicted binding pocket for NS-associated SHP2/E76D mutant (PDB ID: 6CMR); **(C)** Predicted binding pocket for LS-Associated SHP2/T468M mutant (PDB ID: 4OHL); **(D)** Predicted binding pocket for SHP2 wild type (PDB ID: 4DGP).

Three 3D protein structures of PTPN11 including wild type (PDB ID: 4DGP), LS-Associated SHP2/T468M mutant (PDB ID: 4OHL), and NS-associated SHP2/E76D mutant (PDB ID: 6CMR) were used. The crystal structure of PTPN11 consisted of N- and C-terminal SH2 domains, and a PTP domain (**Figure 6A**). The mutated amino acids (i.e., E76D for NS and T468M for LS) were located at the PTP domain. Only the crystal structure of 6CMR contained the ligand SHP099. Thus, the binding sites of the other two crystal structures (i.e., 4DGP and 4OHL) can be considered as speculative. Herein, we employed SiteMap implemented in the Schrodinger suite to identify potential ligand binding sites (Halgren, 2007). The top five binding sites for each protein structure were predicted (see **Supplementary Figure S3**) and the binding sites with highest SiteMap scores were selected for further analysis. The predicted binding site of 6CMR (NS) covered the existing ligand SHP099, validating the performance of SiteMap (**Figure 6B**). The optimal binding sites for 4OHL (LS) and 4DGP (wild type) are illustrated in **Figures 6C, D**, respectively. It was observed that the predicted binding sites of mutated PTPN11 (i.e., 6CMR and 4OHL) shifted from Helix αB to Helix αA of C terminal-SH2 when compared to that of the wild type. The depth between the binding site of NS to the mutated site (E76D) was extended from wild type (17.1 Å) to the mutant state (28.0 Å). However, the distance between the binding site of LS and the mutated site (T468M) was shortened from 24.3 Å to 20.7 Å. Furthermore, hydrophobic areas within mutated structures (i.e., 40HL and 6CMR) were decreased from 95.0 Å to 62.2 Å and 62.1 Å compared to wild type, suggesting conformation changes from wild type to mutated states (**Table 2**). The key residual entropy of mutated structures such as 4OHL and 6CMR varied. For example, we observed that the hydrophobic area of 4OHL (LS) consisted of the residuals *PHE113*, *PRO215*, *LEU216*, *LEU233*, *LEU236*, *LEU254*, and *PRO491* and the hydrophobic area of 6CMR (NS) consisted of the residuals *PHE113*, *LEU117*, *LEU125*, *LEU233*, *LEU236* and *ALA237* (**Table 3**).

**Table 2:** Binding site optimization by SiteMap.

PDB ID	Volume of Pocket	Surface Area	Hydrophilic area (surface area %)	Hydrophobic area (surface area %)	Distance to mutated site E76D for LS	Distance to mutated site T468M of NS
***Wild type of SHP2***						
**4DGP**	222.6	1413.1	1069.4 (75.7%)	95.0 (6.7%)	17.1	24.3
***LEOPARD Syndrome-Associated SHP2/T468M mutant***						
**4OHL**	391.0	1442.5	1066.6 (73.9%)	62.2 (4.3%)	–	20.7
***Closed structure of active SHP2 mutant E76D bound to SHP099 inhibitor for Noonan syndrome***						
**6CMR**	358.1	1325.6	875.3 (66.0%)	62.1 (4.7%)	28.0	–

**Table 3:** Key residuals located at the optimal binding pocket.

PDB ID	Binding type of residuals				
	Hydrophobic	Polar	Negative charge	Positive charge	Not classified
***Wild type of SHP2***					
**4DGP**	PHE7 PRO9 CYS104 PRO107 TRP112 PHE135 VAL194 PRO215 CYS259	SER3 SER109 THR191 GLN214 THR253 GLN255 GLN256 GLN257	ASP106 GLU110 GLU195 GLU252	ARG4 ARG5 ARG111 LYS198 LYS199 LYS213 LYS260	–
***LEOPARD Syndrome-Associated SHP2/T468M mutant***					
**4OHL**	PHE113 PRO215 LEU216 LEU233 LEU236 LEU254 PRO491	THR108 SER109 HIS114 HIS116 ASN217 THR218 THR219 GLN245 THR253	GLU110 GLU232 GLU249 GLU250 GLU252	ARG111 ARG229 LYS492	GLY246
***Closed structure of active SHP2 mutant E76D bound to SHP099 inhibitor for Noonan syndrome***					
**6CMR**	PHE113 LEU117 LEU125 LEU233 LEU236 ALA237	THR108 HIS114 HIS116 THR218 THR219 SER234 GLN245	GLU139 GLU232 GLU238 GLU249 GLU250	ARG229 ARG231 LYS235	GLY115 GLY246

#### Adjusted IFD Model

The Schrödinger Induced Fit Docking (IFD) protocol was implemented to dock the repositioning candidates to the optimal binding site of 4DGP (wild type), 4OHL (LS), and 6CMR (NS), respectively. Some repositioning candidates (e.g., sirolimus, dactinomycin, atracurium besilate, rifabutin, tacrolimus, and asiaticoside for NS, and carbenoxolone, cyanocobalamin, and oxamic acid for LS) failed to dock into protein binding pockets. Building on this, compounds with positive IFD score values were also considered as docking failures. Overall, the success in the execution of IFD to generate repositioning candidates (e.g., 61 for NS and 43 for LS) resulted in multiple docking poses for each repositioning candidate. We selected the best docking pose based on the lowest IFD score for each repositioning candidate. We implemented an adjusted IFD score strategy by combining the IFD scores of ligands in both the mutated and wild type (see *Materials and Methods* section). We found that the IFD scores of LS were higher than that of their wild type, while the IFD scores of NS were lower than their wild type (see **Supplementary Table S6**) suggesting that the stability of protein-ligand conformations for LS is lower than for NS. The adjusted IFD scores were used to reflect the specificity of repositioning candidates binding to the mutated protein structures. However, the prioritization of compounds based on adjusted IFD scores should generally conform to the original IFD approach. Therefore, we further compared the correlation among IFD scores for mutant structures and adjusted IFD scores (see **Supplementary Figure S4**). The Pearson's correlation coefficients between adjusted IFD scores and IFD scores for NS and LS were in the range of 0.883 to 0.982, suggesting the ranking of repositioning candidates was well preserved with only compounds with low binding specificity being excluded.

**Figure 7:**
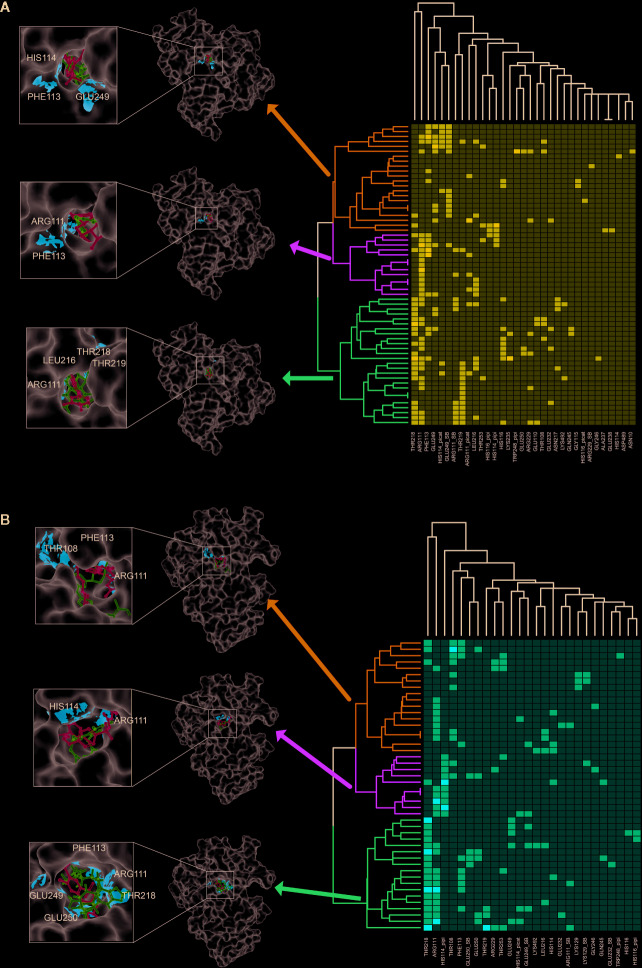
Hierarchical cluster analysis (HCA) of repositioning candidates and binding amino acids for **(A)** NS and **(B)** LS.

To further investigate the key residuals involving in the ligand binding process, we analyzed the ligand-residual binding interactions for 4OHL (LS) and 6CMR (NS) with hierarchical cluster analysis (HCA). For NS, the repositioning candidates were categorized into three clusters based on their binding residuals (**Figure 7A**). Dopamine receptors including BRD-K85818861, levomepromazine, quinpirole, and dopamine for NS were in the same cluster (show in blue). The repositioning candidates in this cluster preferentially bind with residuals such as *HIS114* (polar), *PHE113* (hydrophobic), and *GLU249* (negative charge). Repositioning candidates belonging to Phosphoinositide 3-kinases (PI3Ks) inhibitors (i.e., PI-103 and S1205) and non-selective, irreversible monoamine oxidase (MAOI) inhibitors (i.e., iopromide and iproniazid) were clustered together (shown in red), where more frequently binding residuals included *ARG111* (positive charge), *LEU216* (hydrophobic), *THR218* (polar), and *THR219* (polar). There was another group of repositioning candidates with diverse modes of action (shown in green). The average and standard deviation values of adjusted IFD scores for the three clusters (e.g., -1170.15 ± 4.382 for the blue cluster, -1172.15 ± 3.7695 for the green cluster, and -1171.97 ± 4.6227 for the red cluster) were not statistical significantly different. For LS, repositioning candidates were also divided into three clusters (**Figure 7B**). Most of the histone deacetylase (HDAC) inhibitors (i.e., S1030 and scriptaid) and the BCR-ABL inhibitor (i.e., dasatinib) were clustered together (shown in red). Key residuals such as *PHE113* (hydrophobic), *THR218* (polar), *GLU249/250* (negative charge), and *ARG111* (positive charge) were more frequently bound. Two heat shock protein 90 inhibitors (i.e., radicicol and BRD-K41859756) clustered together (shown in green) and key residuals including *ARG111* and *HIS114* were highlighted. Repositioning candidates that are MOA-dependent formed another cluster (shown in blue). Furthermore, the average adjusted IFD scores of the cluster including heat shock proteins (e.g., -1102.42 ± 14.65) was higher than that of the other two clusters (1111.96± 16.60 for the HDAC cluster and -1110.78 ± 15.18 for the MOA cluster).

#### A Combined Ranking Strategy

To take advantage of both transcriptomic and structure-based approaches for repositioning candidates prioritization, we developed a consensus ranking strategy (see *Materials and Methods* section). The prioritized repositioning list for NS and LS by proposed combined ranking strategy are listed in **Supplementary Table S5**. To further verify the prioritized repositioning list for their potential for LS or NS treatment, real-world evidence including the on-going clinical trials in clinicaltrial.gov and PubMed literature reports were collected. There were very few clinical trial studies focused on the two diseases, and only 1 clinical trial for LS and 13 clinical trials for NS were found. Many of the clinical trials were focused on repositioning candidates despite a lack of literature support. Moreover, the phenotype of NS patients involved in one study was not available in the original publication (Mulero-Navarro et al., 2015). However, LS patients with visible HCM symptoms were described in one initial report (Carvajal-Vergara et al., 2010) and further confirmed with our pathway and network analysis. Altogether, we have mainly focused on PubMed literature reports on the repositioning candidate list for LS to verify our results.

**Table 4** shows the compiled list of selected repositioning candidates for LS with their original designated indications, mode of actions, boxed warnings (BW) status in drug labeling, and real-world repositioning evidence. Of 46 repositioning candidates for LS, we found 11 candidates (24%) with at least one literature report analyzing their potential utility for treating LS or LS-related cardiovascular disorders. Considering LS patients are mainly pediatric, we further evaluated safety profiles of repositioning candidates by assessing the boxed warnings in the label of the approved drug. Notably, 18 of 46 candidates (39.1%) were designated for oncology treatment, suggesting the safety profiles will need to be reevaluated when repositioning these candidates for pediatric uses.

**Table 4:** Selected repositioning candidates for LEOPARD syndrome.

Candidate name	Market status	Boxed Warnings	Pharmacological class	Original indications	Notes	References
**dasatinib**	Approved	No	a BCR-ABL inhibitor	chronic myelogenous leukemia (CML) and acute lymphoblastic leukemia (ALL)	A low dose of dasatinib reversed the expression levels of molecular markers of cardiomyopathy and reduced cardiac fibrosis in NS and LS mice. Low-dose dasatinib may represent a unifying therapy for the treatment of PTPN11-related cardiomyopathies	PMID: 27942593
**scriptaid**	Investigational	–	a histone deacetylase (HDAC) inhibitor	Most early research on anti-cancer activity, cloning and regulation of metabolism	Scriptaid can further be used in the treatment of cardiac/vasculature diseases such as hypertrophy, hypertension, myocardial infarction, reperfusion, ischaemic heart disease, angina, arryhtmias, hypercholestremia, atherosclerosis and stroke	US20140235671A1
**TWS119**	Investigational	–	a glycogen synthase kinase 3 beta (GSK3β) inhibitor	Most early research on anti-cancer activity	Inhibition of GSK3 results in changes in the activities of transcription and translation factors in the heart and promotes hypertrophic responses, and it is generally assumed that signal transduction from hypertrophic stimuli to GSK3 passes primarily through protein kinase B/Akt (PKB/Akt)	PMID: 18204489
**6-bromoindirubin-3-oxime**	Investigational	–	a glycogen synthase kinase 3β (GSK3β) inhibitor	Most early research on anti-cancer activity	The hybrid hydrogel system can co-deliver 6-Bromoindirubin-3-oxime (BIO) and insulin-like growth factor 1 (IGF-1) to areas of myocardial infarction and thus improve cardiac function by promoting the proliferation of cardiomyocytes and revascularization.	PMID: 26251592
**lansoprazole**	Approved	No	a H+/K+-ATPase blocker	peptic ulcer disease, gastroesophageal reflux disease, and Zollinger–Ellison syndrome	Lansoprazole alleviates pressure overload-induced cardiac hypertrophy and heart failure in mice by blocking the activation of β-catenin	PMID: 30689763
**resveratrol**	Approved	No		Herpes labialis infections	there are potential beneficial effects of resveratrol in CVDs (atherosclerosis, hypertension, stroke, myocardial infarction, heart failure)	PMID: 27144581
**nicardipine**	Approved	No	a potent calcium channel blockader	marked vasodilator action	nicardipine may improve left ventricular diastolic function of patients with HCM without serious side effects	PMID: 2636085
**bromocriptine**	Approved	No	a D(2) dopamine receptor	Parkinsonian Syndrome	bromocriptine improves the hemodynamic profile in heart failure acutely	PMID: 6346844
**LY-294002**	Investigational		a strong inhibitor of phosphoinositide 3-kinases (PI3Ks); a BET inhibitor	Most early research on anti-cancer activity	PI3K inhibition by LY294002 or wortmannin abolished the myocardin-dependent SRF stimulation observed in the T468M LS cell line. Consequently, these data suggest that LS-induced PI3K upregulation can enhance SRF/myocardin activity in a reconstituted model.	PMID: 20308328
**glycocholic acid**	Investigational		a crystalline bile acid		bile acids (Bas) as newly recognized signaling molecules that modulate cardiovascular function	PMID: 21707953
**glibenclamide**	Approved	No	an ATP-sensitive potassium channels inhibitor	type 2 diabetes	Glibenclamide may increase the probability of spontaneous termination of ventricular fibrillation and facilitate the restoration of the myocardial function during regional ischaemia.	PMID: 1291084

## Discussion

There is still an extensive unmet medical need in the treatment of rare diseases such as NS and LS. Considering the limited knowledge and availability of data on rare diseases, we pursued a “fit-for-purpose” strategy for exploring treatment opportunities. In this study, we proposed a hybrid strategy to integrate transcriptomic and structure approaches to look for respective repositioning candidates for NS and LS. We found the transcriptomic signatures could discriminate the two diseases despite their high pathological similarity. Furthermore, an adjusted IFD model was able to take into consideration the binding behaviors of both mutated and wild types to reflect the binding specificity. Some key residuals involved in the ligand and mutated 3D PTPN11 binding were highlighted, which could be used for further repositioning candidate enrichment or the development of novel lead compounds. Also, a comparative ranking selection was developed to prioritize repositioning candidates. Finally, a list of repositioning candidates including 61 for NS and 43 for LS was proposed and verified in a literature survey.

Development of the tyrosine phosphatase SHP2 (PTPN11) inhibitors has been the focus of considerable research efforts in light of their diverse role in cancer and RASopathies disorders (Butterworth et al., 2014; Garcia Fortanet et al., 2016; Yu and Zhang, 2018). However, there is a remaining challenge around selectivity for specific phenotypic endpoints and cell permeability of potential inhibitors. The well-established approaches for PTPN11 inhibitor identification mainly rely on *in silico* and *in vitro* screening against large compound repositories. However, uncertainty remains regarding the potential adverse effects of the enriched lead compounds. The proposed hybrid approach is helpful to better understand the perturbation by of different biological process at the molecular level. Furthermore, the adjusted IFD model aims to prioritize the compounds based on their binding specificity to the mutated protein structure, which may increase the selectivity of compounds for treating more precise disease endpoints, further eliminating unexpected side effects.

A large proportion of enriched repositioning candidates for LS and NS has anticancer activity. In our previous study, we have proposed to reuse oncology drugs for rare disease treatment development based on the similarity of the two types of diseases at the molecular level (Liu et al., 2016). The mutation of the PTPN11 gene is not the only causative link to NS and LS but is also one of the most somatic mutated genes (SMGs) in cancer pathogenesis (Kandoth et al., 2013). For example, RASopathies disorders such as NS and LS are correlated with specific cancer types such as Juvenile Myelomonocytic Leukemia (JMML), attributed to different mutation types of the PTPN11 gene (Kratz et al., 2005; Kiel and Serrano, 2014). One of the proposed repositioning candidates for LS was dasatinib, a BCR-ABL inhibitor, that was originally designed for treating chronic myelogenous leukemia (CML) and acute lymphoblastic leukemia (ALL). It was reported that a low dose of dasatinib reversed the expression levels of molecular markers of cardiomyopathy and reduced cardiac fibrosis in NS and LS mice with the PTPN11 mutation (Yi et al., 2016).

One of the most common phenotypic characteristics of NS and LS is hypertrophic cardiomyopathy (HCM). The LS patients involved in this study had a clear HCM phenotype. Multiple small molecule inhibitors of histone deacetylase (HDAC) have been shown to be efficacious in blocking pathological cardiac hypertrophy, and in improving cardiac function in various rodent animal models (Bush Erik and McKinsey Timothy, 2010; Ferguson et al., 2013). Several HDAC inhibitors including scriptaid, KM 00927, and S1030 emerged in our repositioning candidate list for LS treatment. For example, one patent described the use of scriptaid in the treatment of cardiac/vasculature diseases such as hypertrophy, hypertension, myocardial infarction, and ischaemic heart disease (US20140235671A1).

Our data also suggested several heat shock protein 90 (HSP90) inhibitors including BRD-K41859756, geldanamycin, radicicol for potentially treating LS. HSP90 has been widely studied given its essential role in cancer etiology (Wu et al., 2017). Alongside this, the cardiac protection function of HSP90 has also been described. For example, induction of HSP90 by mild stress has a protective effect against severe heart pressure. Furthermore, over-expression of HSPs in cardiac cells in transgenic animal and virus models suggest a potential route to the development of treatments to protect heart function (Latchman, 2001).

Several areas would benefit from further verification of the strategies and results suggested from this study. First, due to the limited samples and data of NS (e.g., one sample with two replicates), the transcriptomic signatures may suffer a degree of bias leading to unspecific functional analysis results. Second, the adjusted IFD model is time-consuming due to the need for precise docking for both mutated and wild types, limiting its broader application for screening purposes. However, the adjusted IFD model could easily be migrated to the Glide docking framework to address this limitation. Finally, no single bioinformatics approach offers a one-stop solution for exploring rare disease treatment options. Each research question requires a “fit for purpose” approache based on biological understanding and data availability. More importantly, the prioritized repositioning list serves as a starting point for further treatment development, and wet lab experiment verification and real-world evidence are imperative (Delavan et al., 2018).

It is worthwhile to consider additional experimental studies to confirm the findings from our study. The current study set the focus on the novel bioinformatics approaches by integrating genomics and structure information to generate testable hypotheses and provide viable leads for other investigators to follow up on. However, the clinic validity of enriched candidates could be only established with further wet-lab experiments and clinical trials, which is out of the scope of the current study. Specifically, a more profound transcriptome analysis should be conducted to show that candidate drugs might influence the transcriptome in such a way that the NS and LS cells could come back to normal profile. Moreover, hiPS cells with NS and LS syndromes should be analyzed for cardiac defects. Then, the proposed drugs should be demonstrated to functionally rescue these defects. Furthermore, some experimental validation of the cellular activity of the proposed drugs against the disease targets (e.g., PTPN11) could be helpful to elucidate the pharmacological effects of enriched candidates. In our future work, we will further consider how to enhance biological validation for a better verification of proposed in silico approaches.

In conclusion, we have developed a computational drug repositioning framework that integrates transcriptomic and structural approaches to find novel potential NS and LS treatment options. The framework developed is robust and relatively straightforward to use as such it may be equally applicable to the search for treatments for other rare diseases.

## Materials and Methods

### Transcriptomic Profiles of Noonan and LEOPARD Syndrome

The transcriptomic profiles of NS and LS were extracted from Gene Expression Omnibus (GEO, https://www.ncbi.nlm.nih.gov/geo/) by using keyword “*Noonan syndrome*” AND (“*LEOPARD syndrome*” OR “*Noonan syndrome with multiple lentigines*”). Consequently, two GEO datasets GSE54538 (Mulero-Navarro et al., 2015) and GSE20473 (Carvajal-Vergara et al., 2010) were obtained for NS and LS, respectively. Interestingly, the transcriptomic profiles of the two studies were both generated based on samples derived from human iPSC (**Table 1**).

The samples of NS dataset (GSE54538) included NS patients, juvenile myelomonocytic leukemia (JMML) patients, NS/JMML patients, and healthy donors. Here, NS patient samples with E76D mutation in PTPN11 (i.e., one patient with two biological replicates) and four control samples from healthy donors were employed for further analysis.

The experiment samples of LS (GSE20473) consisted of two iPSC lines of two LS patients with T468M mutation in PTPN11, normal or patient Fibroblasts, and HES2 human ES cell line. In this study, two iPSC lines from each LS patient (i.e., four samples: L1-iPS1, L1-iPS13, L2-iPS6, and L2-iPS16) and two controls including the HES2 human ES cell line and a wild-type iPSC line and BJ-iPSB5 derived from a normal human fibroblast line (BJ) was used.

### Microarray Data Normalization and Disease Signatures Generation

For NS dataset (GSE54538), Illumina HumanHT-12 V4.0 expression BeadChip was used, which targets more than 47,000 probes derived from the National Center for Biotechnology Information Reference Sequence (NCBI) RefSeq Release 38. The preprocessed data by Robust Multi-array Average (RMA) was downloaded from https://www.ncbi.nlm.nih.gov/geo/query/acc.cgi?acc=GSE54538. By comparing NS patient samples to the controls, the fold changes and *p* values were generated using R *limma* package. We further mapped the probe sets into gene symbols using R *biomaRt* packages. Then, the probes that could not be converted to gene symbols were excluded. Final, we ranked genes based on their fold change values and selected the top/down 250 genes as NS signatures (see **Supplementary Table S1**).

For LS dataset (GSE20473), the experiment was conducted using Affymetrix Human Exon 1.0 ST Array. The Raw CELL files were downloaded from https://www.ncbi.nlm.nih.gov/geo/download/?acc=GSE20473&format=file. First, the RAW CELL files were processed with alternative chip definition files (CDFs, Version 19.0.1, ENTREZG) from Brainarray (http://brainarray.mbni.med.umich.edu/Brainarray/Database/CustomCDF/CDF_download.asp) and RMA used to summarize the intensity ratios at probe set level to obtain expression values per gene. Like the procedure used for NS data, the fold change and *p* values were generated by using R limma and limited to gene levels. Finally, genes were ranked by their fold changes values, and the top/down 250 genes were extracted as the LS signatures (see **Supplementary Table S1**).

### Pathway Analysis

The Kyoto Encyclopedia of Genes and Genomes (KEGG) pathway analysis was carried out with the obtained disease signatures including 500 genes (i.e., top/down 250 genes) for NS and LS by using the Database for Annotation, Visualization and Integrated Discovery (DAVID, version 6.8) software (Huang et al., 2008). The pathways with a Benjamini-Hochberg (BH) adjusted *p*-value less than 0.05 were considered to be statistically significant.

### Protein-Protein Interaction (PPI) Network Analysis

Protein-protein interactions (PPI) among NS and LS signatures were further determined by human PPI data extracted from the STRING v11.0 database (https://string-db.org/) (Szklarczyk et al., 2017). Only the interactions with high confidence interaction scores defined by the STRING database (e.g., ≥ 0.7) were considered. We further extracted the sub-networks by using the MCODE plug-in for Cytoscape (version 3.7.1), which is designed to expand the cluster from highly interconnected seed nodes by setting a certain threshold (Bader and Hogue, 2003).

### miRNA Target Prediction

To further investigate the distribution of miRNA regulating NS and LS signatures, a consensus of twelve *in silico* miRNA prediction algorithms including miRWalk, miRDB, PITA, MicroT4, miRMap, RNA22, miRanda, miRNAMap, RNAhybrid, miRBridge, PICTAR2, and Targetscan were employed. We considered the mRNA-miRNA relationships that were predicted as positive by 10 of 12 algorithms. The calculation was carried out through miRNA 2.0 (http://zmf.umm.uni-heidelberg.de/apps/zmf/mirwalk2/miRretsys-self.html) (Dweep et al., 2011).

### Phenotypic Anchoring

One of the most common phenotypes of NS and LS are cardiovascular disorders such as hypertrophic cardiomyopathy (HCM). We collected four transcriptomic profiles of HCM (i.e., GSE89714, GSE68316, GSE36961, and GSE32453) by searching against the GEO database (see **Table 1**). The differentially expressed genes (DEGs) were calculated for each dataset by comparing patient group to the control group. We then compared the NS and LS transcriptomic signatures to the DEGs of HCM, respectively. All the calculations were carried out with R *limma* packages.

### Repositioning Candidate Enrichment by Using CMap and LINCS L1000

The hypothesis behind genome-based repositioning is that if the drug signature is reversely correlated with the disease signature, the drug could be potentially used to treat the diseases. Here, two drug-induced transcriptional profiles of human cell lines including NIH LINCS project (http://www.lincsproject.org/) (Subramanian et al., 2017) and Connectivity Map 02 (Lamb et al., 2006) were employed to enrich the repositioning candidates for NS and LS, respectively.

Specifically, LINCS L1000 characteristic direction signatures search engine (L1000CDS^2^) was used to reversely compare the NS and LS transcriptomic signatures to the drug transcriptional signatures in LINCS project for repositioning candidate enrichment. The top 50 candidates based on cosine similarity scores were extracted as the repositioning candidates. For CMap 02 dataset, 6,100 experiments were conducted in six or seven different cancer cell lines using 1,309 compounds with two Affymetrix platforms. We followed the standard procedures to process and normalize the dataset (Lamb et al., 2006). To eliminate the divergence among cancer cell lines, we merged multiple experiments for the same compounds into a single Prototype Ranked List (PRL) following the processing described elsewhere (Iorio et al., 2010). Then, Gene Set Enrichment Analysis (GSEA) scores for measuring the reverse correlation degree between NS/LS transcriptomic signatures and drug PRL was calculated following the strategies described in previously (Sirota et al., 2011). The top 50 compounds by ranked GSEA score were used as repositioning candidates.

To further investigate whether the enriched compounds are by chance, an equal number of pseudo-NS and -LS transcriptomic signatures were generated by randomly selecting 500 genes (i.e., top/down 250 genes) for each drug-transcriptomic data set. Then, the pseudo enrichment scores were calculated. This process was repeated N= 50,000 to remove the potential bias in the gene selection process, and a null distribution of pseudo enrichment scores was obtained. Finally, the *p*-value for each drug was calculated based on the null distribution.

### Molecular Docking

Repositioning candidates obtained from the transcriptomics-based approach were further prioritized by using molecular docking. Since the patients involved in the transcriptomic studies of NS and LS has PTPN11 mutants (i.e., T468M for NS and E76D for LS, respectively), the 3D structures of PTPN11 including wild type, T468M mutation, and E76D mutation were retrieved from RCSB PDB (Yang et al., 2018). The 3D crystal structures including PTPN11 wild type (PDB ID: 4DGP), mutant T468M (PDB ID: 4OHL), and mutant E76D (PDB ID: 6CMR) were downloaded. The structure of PTPN11 with E76D mutation (PDB ID: 6CMR) included a ligand SHP099 inhibitor.

Molecular docking was carried out by Schrödinger (Schrödinger, LLC, New York, NY, 2018). All protein structures were prepared with Protein Preparation Wizard in the Schrödinger Suite. The structural integrity was checked, and missing site chains and loops segments were added using Prime. Hydrogen atoms were added without deleting the original ones. All ligands of repositioning candidates based on transcriptomics-based approach were prepared with LigPrep (LigPrep, Schrödinger, LLC, New York, NY, 2018) in the Schrödinger Suite with the default settings. The four poses with minimal energy were kept.

The potential binding sites of the 3D structure of mutated and wild type PTPN11 were predicted by the SiteMap program in Schrödinger software. The predicted binding sites for the PTPN11 with E76D mutations were compared with the pocket where the SHP099 inhibitor was located. The binding site with the highest score was selected as the most probable binding sites for further docking.

Induced fit docking (IFD) predicts optimal ligand-binding modes and concomitant structural movements in the receptor using Glide and Prime modules. In IFD, when a ligand binds to the receptor, it undergoes side-chain or backbone conformational changes or both in many proteins. These conformational changes permit better binding to the receptor according to the shape and binding mode of the ligand. Docking was performed using the Schrödinger Induced Fit Docking (IFD) protocol, consisting of GLIDE docking with the rigid protein followed by energy minimization of the side chain orientations with Prime of those residues within 7.5 Å of the ligand. In the first Glide docking round, the ring containing carbons 1, 2, 3, 4, 5, and 10, and the hydroxyl at C3 of the estradiol core were restricted to an RMSD of 1.0 Å from the crystal pose. A final round of GLIDE docking was then performed towards the optimized receptor structures, from which final Induced Fit Docking (IFD) scores for each ligand-receptor complex were calculated.

We developed an adjusted IFD score to reflect the specificity of compounds binding to the structure of mutated SHP-2 with the following equation:

(1) $$adjusted\;IFD\;score_{i}=IFD\;score_{mutated,i}+c$$

(2) $$c=(\max(IFDscore_{mutated,n})-min(IFDscore_{mutated,n}))\times \frac{IFD\;score_{mutated,i}-IFD\;score_{wild\;type,i}}{\max(IFD\;score_{wild\;type,n})-\min\;(IFDscore_{wild\;type,n}}\;i,n\;\in 1,2,3,\ldots ,50$$

Where *c,* the adjusted parameter, is normalized the difference of IFD scores between mutated and wild type protein structures for compound *i*. The *c* was used to measure the specificity of compound binding to mutated PTPN11 structure.

### Consensus Ranking Strategy

To combine the transcriptomic-based approach with molecular docking for generating a consensus ranking list, we developed a combined score as listed below,

(3) $$Combined\;score_{i,CMap/L1000}=\;\;\frac{Enrichment\;score_{Cmap,i/L1000,i}+Normalized\;adjust\;IFD\;score_{i}}{2}$$

(4) $$Normalized\;adjust\;IFD\;score_{i}=\frac{\left|adjusted\;IFD\;score_{i}|-\min(|adjusted\;IFD\;score_{n}|\right)}{\max(|adjusted\;IFD\;score_{n}|)-\min(|adjusted\;IFD\;score_{n|})}\;i,n\;\in 1,2,3,\ldots ,50$$

## Data Availability Statement

Publicly available datasets were analyzed in this study. This data can be found here: GSE54538; GSE20473;GSE89714;GSE68316; GSE36961; GSE32453.

## Author Contributions 

ZL and WT conceived and designed the study. LZ and ZL performed data analysis and wrote the manuscript. RH, MX, and JZ designed and conducted the wet-lab experimental verification. LZ, RR, BD, RH, ZL, and WT revised the manuscript. All authors contributed to the article and approved the submitted version.

## Disclaimer

The views presented in this article do not necessarily reflect current or future opinion or policy of the U.S. Food and Drug Administration and/or the U.S. National Institute of Health. Any mention of commercial products is for clarification and not intended as an endorsement.

## Conflict of Interest

RR is co-founder and co-director of ApconiX, an integrated toxicology and ion channel company that provides expert advice on non-clinical aspects of drug discovery and drug development to academia, industry and not-for-profit organizations.

## Supplementary Material

Figure S1 Venn diagram of consensus predicted miRNAs for Noonan syndrome (NS) and LEPOARD syndrome (LS) transcriptomic signatures

Figure S2 Venn diagram of Noonan syndrome (NS)/LEPOARD syndrome (LS) transcriptomic signatures and differentially expressed genes (DEGs) of four independent hypertrophic cardiomyopathy (HCM) patient-related whole genome gene expression data sets. **(A)** Commonality of up-regulated of genes among NS and four independent HCM patient-related transcriptomic studies; **(B)** Commonality of up-regulated of genes among LS and four independent HCM patient-related transcriptomic studies; **(C)** Commonality of down-regulated of genes among NS and four independent HCM patient-related transcriptomic studies; **(D)** Commonality of down-regulated of genes among LS and four independent HCM patient-related transcriptomic studies.

Figure S3 Top five binding sites of 3D SHP2 protein structures of Noonan syndrome (NS), LEPOARD syndrome (LS) and wild type by using Schrodinger SiteMap module. **(A)** NS; **(B)** LS; **(C)** wild type.

Figure S4 Correlation between Induced Fit Docking (IFD) and adjusted IFD scores of repositioning candidates for Noonan syndrome (NS) and LEPOARD syndrome (LS). **(A)** Repositioning candidates for LS enriched from L1000 dataset; **(B)** Repositioning candidates for LS enriched from CMap dataset; **(C)** Repositioning candidates for NS enriched from L1000 dataset; **(D)** Repositioning candidates for NS enriched from CMap dataset.
