# Supplementary figures and images for: Drug Repositioning for Noonan and LEOPARD Syndromes by Integrating Transcriptomics With a Structure-Based Approach
Source: Front Pharmacol. 2020 Jun 26;11:927. doi: 10.3389/fphar.2020.00927 (PMC7333460; doi:10.3389/fphar.2020.00927)

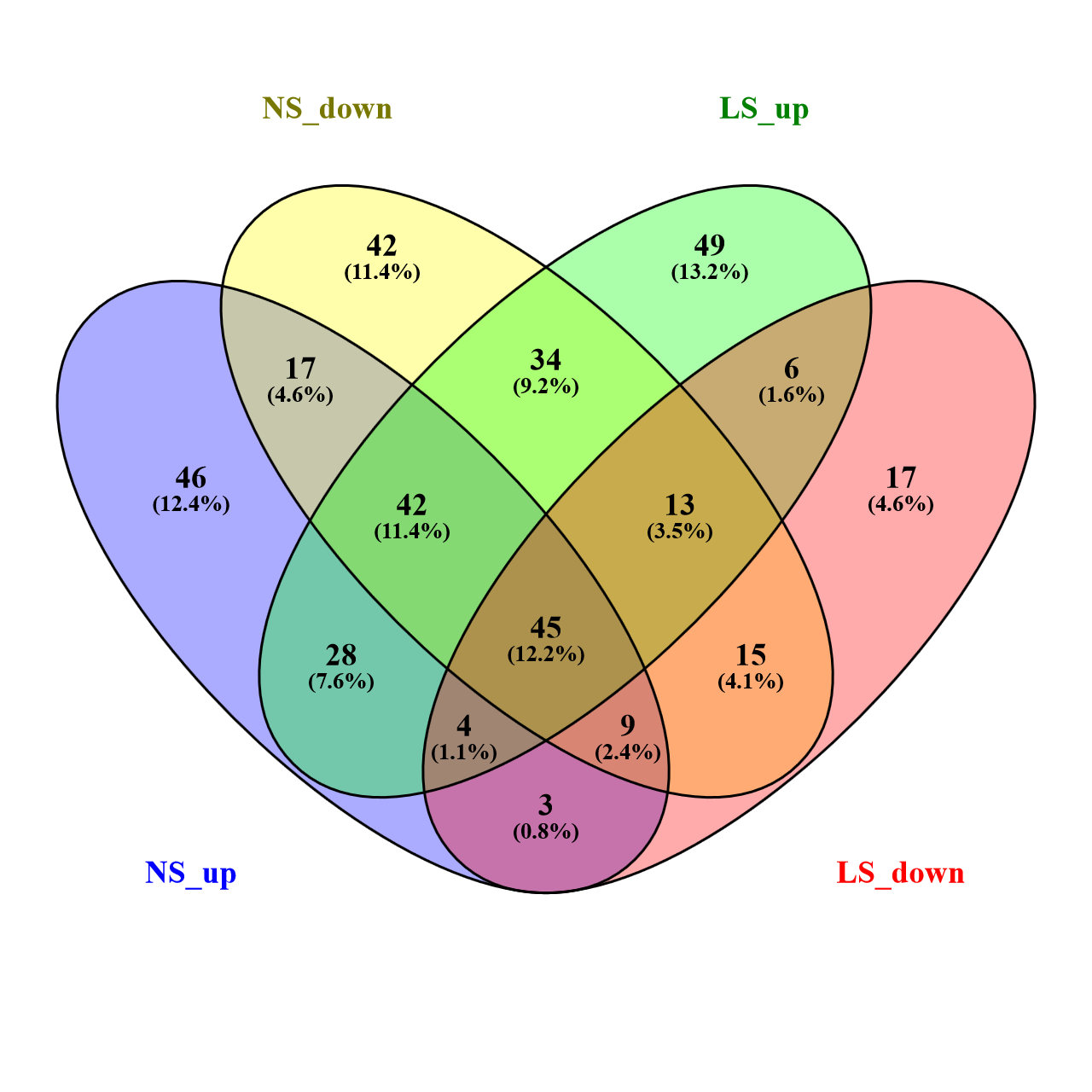

Supplement: Figure S1 — Venn diagram of consensus predicted miRNAs for Noonan syndrome (NS) and LEPOARD syndrome (LS) transcriptomic signatures [file Image_1.png]

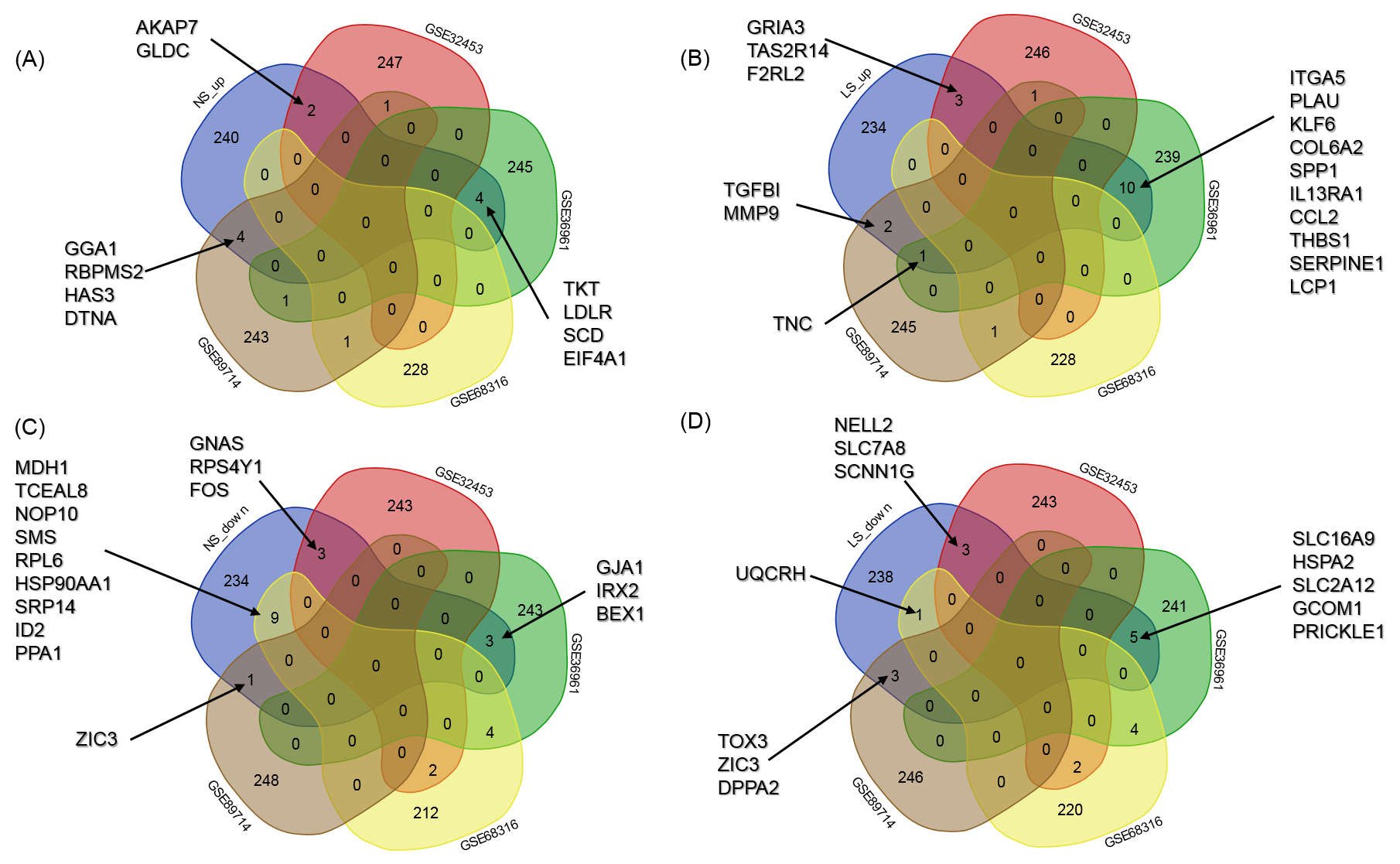

Supplement: Figure S2 — Venn diagram of Noonan syndrome (NS)/LEPOARD syndrome (LS) transcriptomic signatures and differentially expressed genes (DEGs) of four independent hypertrophic cardiomyopathy (HCM) patient-related whole genome gene expression data sets. (A) Commonality of up-regulated of genes among NS and four independent HCM patient-related transcriptomic studies; (B) Commonality of up-regulated of genes among LS and four independent HCM patient-related transcriptomic studies; (C) Commonality of down-regulated of genes among NS and four independent HCM patient-related transcriptomic studies; (D) Commonality of down-regulated of genes among LS and four independent HCM patient-related transcriptomic studies. [file Image_2.png]

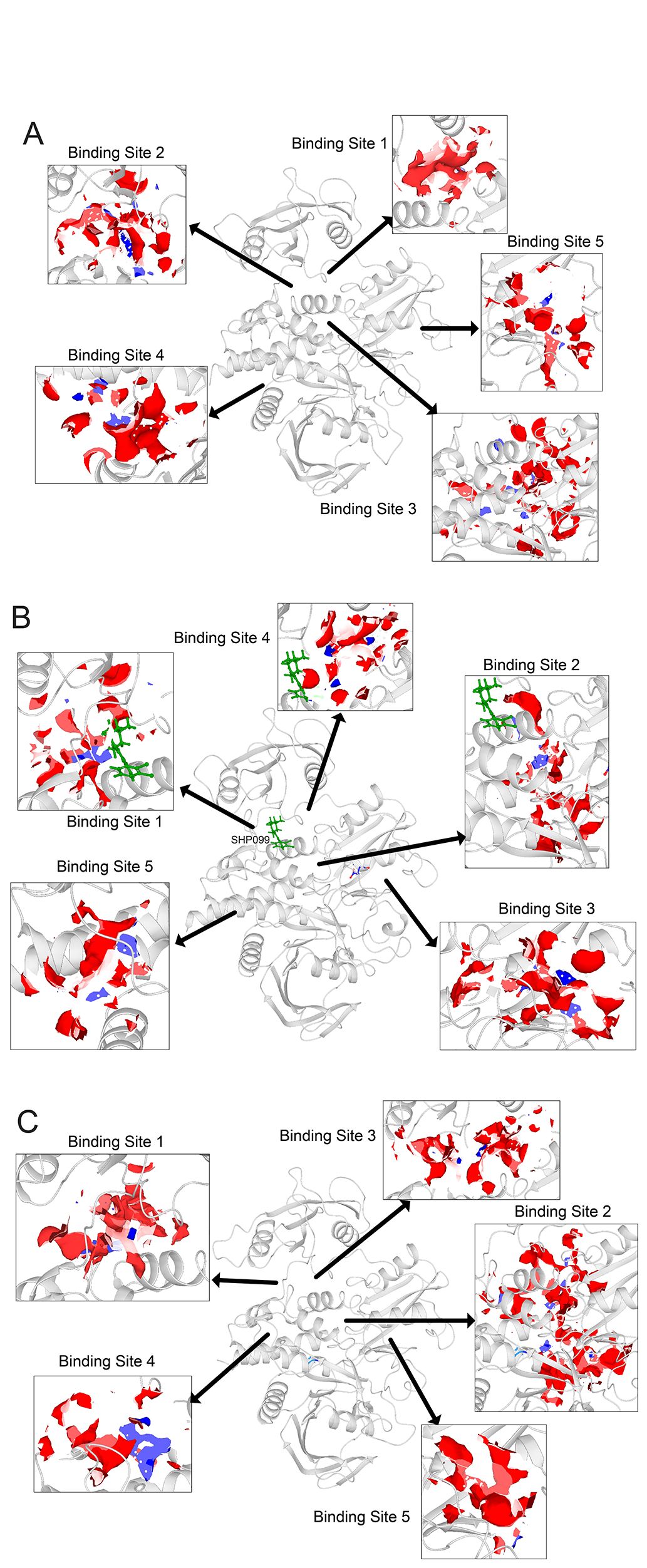

Supplement: Figure S3 — Top five binding sites of 3D SHP2 protein structures of Noonan syndrome (NS), LEPOARD syndrome (LS) and wild type by using Schrodinger SiteMap module. (A) NS; (B) LS; (C) wild type. [file Image_3.tif]
